# The bacterial aetiology of adult community-acquired pneumonia in Asia: a systematic review

**DOI:** 10.1093/trstmh/tru058

**Published:** 2014-04-29

**Authors:** Leon Peto, Behzad Nadjm, Peter Horby, Ta Thi Dieu Ngan, Rogier van Doorn, Nguyen Van Kinh, Heiman F. L. Wertheim

**Affiliations:** a Wellcome Trust Major Overseas Programme, Oxford University Clinical Research Unit, Hanoi and Ho Chi Minh City, Vietnam; b Centre for Tropical Medicine, Nuffield Department of Clinical Medicine, Oxford University, Oxford OX3 7LJ, UK; c National Hospital for Tropical Diseases, Hanoi, Vietnam

**Keywords:** Asia, Community-acquired, Microbiology, *Mycobacterium tuberculosis*, Pneumonia, *Streptococcus pneumoniae*

## Abstract

**Background:**

Community-acquired pneumonia (CAP) is a major cause of adult mortality in Asia. Appropriate empirical treatment depends on knowledge of the pathogens commonly responsible. However, assessing the aetiological significance of identified organisms is often difficult, particularly with sputum isolates that might represent contamination with oropharyngeal flora.

**Methods:**

A systematic review of all adult CAP aetiology studies from Asia, excluding the Middle East, published in English between 1 January 1990 and 1 March 2012 was conducted. Forty-eight studies reporting on 10 423 patients were included, representing data from China, India, Indonesia, Japan, Malaysia, The Philippines, Singapore, South Korea, Taiwan, Thailand and Vietnam. Data from large parts of Asia were unavailable and there was substantial heterogeneity in methodology.

**Results:**

As in western studies, *Streptococcus pneumoniae*, *Mycoplasma pneumoniae*, *Chlamydophila pneumoniae*, *Legionella* spp. and *Haemophilus influenzae* were all significant pathogens. However, compared with western studies, *S. pneumoniae* was of less relative importance. Gram-negative bacilli and *Mycobacterium tuberculosis* were more important, and in northeast Thailand *Burkholderia pseudomallei* was a major pathogen.

**Conclusion:**

These data have major implications for diagnostic strategies and empirical treatment. Narrow-spectrum antibiotics targeting *S. pneumoniae* may be inappropriate in many Asian settings, and agents active against TB may lead to partial response and delayed TB diagnosis.

## Introduction

### Background

Community-acquired pneumonia (CAP) is one of the most common life-threatening infections, with most deaths occurring in developing countries.^1^ The main burden of disease is in children, but CAP is an important cause of mortality in adults, especially the elderly and those with chronic diseases. Antibiotic treatment of CAP is usually empirical as the causative pathogen is rarely identified, even among hospitalised patients in developed countries, and almost never in time to direct immediate treatment. Appropriate selection of empirical treatment depends on the common pathogens identified in previous aetiological studies and on relevant treatment trials. The aetiology of pneumonia is well documented in developed countries (Europe, North America, Japan, Australia), with around 10 bacterial species regularly identified as pathogens in immunocompetent patients. A review of 41 European studies found that *Streptococcus pneumoniae* was by far the most common bacterial cause of CAP, followed by *Mycoplasma pneumoniae*, *Chlamydophila pneumoniae*, *Legionella pneumophila* and *Haemophilus influenzae*.^2^

In Asia, CAP is estimated to cause almost one million adult deaths per year. Many of these deaths occur in the elderly, but a large number occur in those with good life expectancy, including 160 000 among those aged 15–59 years.^3^ However, adult CAP has been poorly studied in Asia until recently. Although differences between these recent studies and European data have been noted,^4–6^ there has been no systematic review of CAP aetiology in Asia. Knowledge of local aetiology is critical to making rational decisions about empirical antibiotic treatment, as differences in aetiology may result in poor response to therapy chosen to cover pathogens common in western studies. Both under-use and over-use of broad-spectrum antibiotics for an infection as common as CAP could be harmful, particularly in Asia where mortality is high, resources scarce and antibiotic resistance an increasing problem.^7,8^

The aim of this systematic review was to synthesise the results of CAP aetiology studies performed among adults in Asia, focusing on bacterial pathogens. This review includes an assessment of study methodology and highlights geographical differences in aetiology, identifying areas requiring further research.

### Identifying the aetiology of pneumonia

Identification of the pathogens responsible for pneumonia is challenging due to difficulties obtaining direct lung samples as well as the oropharyngeal contamination of expectorant. Interpreting the results of pneumonia aetiology studies requires an understanding of the limitations thereby imposed. This has been reviewed elsewhere and is discussed briefly here.^9^

Taking samples directly from the lung through transthoracic needle aspiration represents a theoretic ideal diagnostic method, with high rates of positive results. Similar high-quality results can be obtained through some bronchoscopic techniques. These procedures are often considered too invasive, with costs and risks outweighing perceived benefit. Blood culture is a common procedure in clinical practice, and bacterial growth from blood is almost certainly significant, however the rate of positive culture is typically <10%.^10^ Pleural effusions can be tapped safely, when they are present; however, the sensitivity of pleural fluid culture is poor.

A higher rate of positive culture is obtained from sputum, although contamination by bacteria colonising the oropharynx makes interpreting the significance of sputum isolates difficult.^11^ Microscopic examination of sputum for the presence of white blood cells and epithelial cells can increase the reliability of sputum culture.^12^ The sensitivity of all microbiological diagnostics relying on culture of living bacteria is hampered by antibiotic use prior to sampling.

Infection by ‘atypical’ bacteria (*Mycoplasma*, *Chlamydophila* and *Legionella* spp.) can be retrospectively assessed through serology; *Legionella* spp. can also be cultured. However, PCR of respiratory specimens is increasingly used. Interpretation of serology is hampered by a lack of standardised techniques and difficulty in distinguishing between current infection and past infection without acute and convalescent samples. Interpretation of PCR is complicated by oropharyngeal contamination and incidental carriage.^13,14^ Urine antigen testing is widely used for two organisms: *S. pneumoniae*, where the test performs well in adults;^15,16^ and *L. pneumophila*, where the test is specific and much quicker than culture but lacks sensitivity, especially in less severe cases.^17^

Retrospective studies of CAP face problems related to uncertainty about case definition, incomplete recording of clinical information and incomplete or poor-quality microbiological investigation. Often these studies have a focus on a particular group of pathogens and rarely employ a broad, systematic approach. Increasingly it is recognised that a control group is helpful where upper airway samples are being taken and colonisation, rather than infection, is a possibility.

## Methods

### Search strategy and selection criteria

To minimise bias associated with retrospective studies, only prospective studies have been included, except as discussed below. The aetiology of CAP varies with clinical setting, therefore studies were grouped into outpatient studies, hospital admissions and studies of severe CAP. These categories are also typically used to decide empirical treatment. Few prospective studies were found for severe CAP, therefore retrospective studies were included for this category.

The search consisted of three components: 1. ‘pneumonia’ or ‘respiratory tract infection*’ in the title; 2. geographical terms anywhere in the citation using names of all countries along with ‘Asia’ and MeSH terms for Central, Southeast and East Asia (Siberia and the Middle East, including Iran, were not included in the review); and 3. limits specifying a publication date after 31 December 1989 up to 1 March 2012. The title and abstract of all retrieved citations were reviewed, and the full text was retrieved if the abstract suggested the article contained data on CAP aetiology in Asia. Case reports, laboratory-based studies and studies on children or other selected subgroups of CAP patients were not reviewed.

The reference lists of all retrieved papers were searched for additional relevant studies. Where necessary, study authors were contacted for further information, including data disaggregated by country from multinational studies. Articles were included in the quantitative review if they were published in English and 1. reported on a prospective cohort of CAP patients or a retrospective cohort of severe CAP patients; 2. had consistent investigation of aetiology and 3. included a clearly selected sample of chest radiography-confirmed CAP, without exclusions that would make the sample non-comparable with other studies. Structured data extraction was performed for all studies included in the quantitative analysis, including patient selection criteria, demographics and scheme of microbiological investigation.

## Results

### Study selection

**Figure 1.**
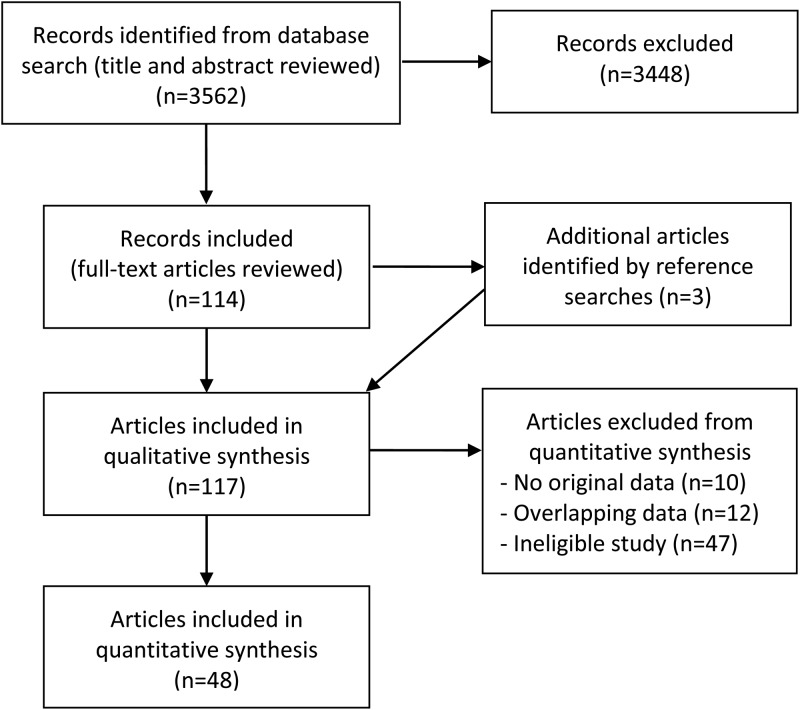
PRISMA flow diagram of study selection.

In total, 3562 citations were retrieved and 114 relevant English language articles were identified after screening the title and abstract. The full text of these were reviewed, identifying a further three articles from reference searching. Of the 117 articles, 48 were included in the quantitative review and 69 were excluded, either because they contained no data or duplicate data or did not fulfil the selection criteria (Figure 1).

### Study characteristics

The 48 articles included reports on 50 cohorts of chest radiography-confirmed CAP in 10 423 patients. Of these, four were prospective outpatient cohorts,^18–21^ 38 were prospective inpatient cohorts^20–57^ and eight were cohorts of patients with severe CAP (six retrospective, and defined either as admission to intensive care unit, presence of acute respiratory distress syndrome or meeting the American Thoracic Society criteria for ‘severe’).^58–65^ Two large inpatient studies were multinational^46,55^ and for these studies data from each country were treated separately. Study size ranged from 22 to 1193 patients, with a median of 106 enrolled patients.

Most studies specified some exclusion criteria, usually related to recent hospital admission, nursing home residence, immunosuppression, lung cancer or terminal illness. The mean age was between 50–70 years for inpatient studies and 30–50 years for outpatient studies. The median proportion of males in the studies was 58%. In most inpatient studies more than one-half of participants had some co-morbidity, the commonest being chronic respiratory disease or diabetes.

**Figure 2.**
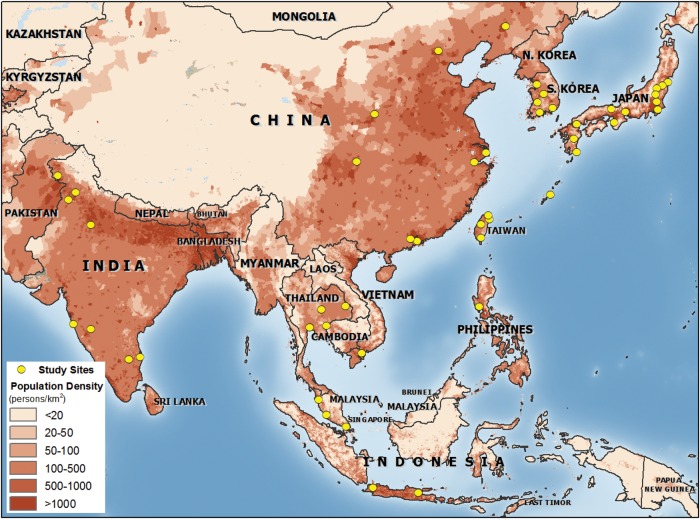
Location of included studies. Map produced by Thanh Le Viet. Software: ArcGIS Desktop 9.3 (ESRI, Redlands, CA). Data sources: population density in 2010, adjusted to match UN totals, persons per square km. (http://sedac.ciesin.columbia.edu/gpw). Country GIS data: www.naturalearthdata.com.

Many countries had no eligible studies, including Bangladesh, Pakistan and the whole of Central Asia, and there was minimal data from Indonesia and Vietnam (Figure 2). Five studies relied only on sputum and blood culture, five tested only for atypical pathogens and the others used a range of methods. Most studies that used sputum culture also reported using some quality criteria to improve reliability, although the criteria used were varied. No studies systematically used transthoracic needle aspiration. The frequency of antibiotic use before admission was reported in 11 studies, ranging from 20 to 64%.

### Aetiology results

The results for individual studies are shown in Tables 1–3. In most studies, between 35 and 70% of participants had no pathogen identified.

**Table 1.** Aetiology of community-acquired pneumonia (CAP) in those admitted to hospital

Country	Year	Site	No. of patients	% *Streptococcus pneumoniae*	% *Haemophilus influenzae*	% *Staphylococcus aureus*	% *Klebsiella pneumoniae*	% Other GNB^a^	% *Mycoplasma pneumoniae*	% *Chlamydophila pneumoniae*	% *Legionella* spp.	% Viruses	% *Mycobacterium tuberculosis*
Japan^34^	1994–1997	Kurashiki	326	23	7	2	4	3	5	3	1	4	2^b^
Japan^20^	1998–2003	Kurashiki	400	26	13	3	2	2	9	7	2	3	–
Japan^51^	1999–2000	Multicentre	232	25	19	3	1	0	5	6	4	16	–
Japan^27^	1999–2002	Fukuoka	227	20	9	7	2	7	7	4	0	4	–
Japan^35^	2001–2004	Kurashiki	349	24/39^c^	6	1	1	2	11	3	1	1	2^b^
Japan^29^	2004–2006	Nagasaki	88	–/18^c^	16	7	0	0	5^b^	–	0	–	–
Japan^31^	2007–2008	Tokyo	102	22/30^c^	8	0	2	4	1	3	1	–	–
Japan^37^	2008–2010	Osaka	170	–/18^c^	4	5	4	14	9	6	0	2^b^	–
Japan weighted average			1894	24/26^c^	10	3	2	4	8	5	1	5	–
South Korea^39^	1999–2000	Chunchon	81	–	–	–	–	–	9	12	0	–	–
South Korea^54^	2001–2002	Multicentre	126	13	1	1	3	10	6	7	2	–	–
South Korea^55,d^	2002–2004	Multicentre	338	14	1	–	3	–	–	–	–	–	–
South Korea weighted average			545	14	1	–	3	–	7	9	1	–	–
Taiwan^38^	2001–2002	Multicentre	168	10/24^c^	5	2	5	2	14	7	1	10	1^b^
Taiwan^46,d^	2001–2002	Taipei	85	–	–	–	–	–	5	6	4	–	–
Taiwan^57^	2001–2002	Kaohsiung	100	22/26^c^	9	1	5	2	20	13	3	1^b^	2
Taiwan^55,d^	2002–2004	Taipei	65	14	2	–	14	–	–	–	–	–	–
Taiwan weighted average			418	14/23^c^	5	1	7	2	14	8	2	–	–
China^24^	1988	Hong Kong	90	9/12^c^	4	4	1	3	3	6	0	6	12
China^46,d^	2001–2002	Hong Kong	62	–	–	–	–	–	2	3	2	–	–
China^32^	2001–2003	Shanghai	314	2	24	2	5	3	12	5	1	–	–
China^55,d^	2002–2004	Multicentre	225	11	9	–	5	–	–	–	–	–	–
China^42^	2003–2004	Multicentre	610	10	9	4	6	3	21	7	5	19^e^	–
China^44^	2004–2005	Hong Kong	1193	8	5	1	1	4	7	5	1^e^	11	3^b^
China weighted average			2494	8/9^c^	9	2	3	3	11	5	3	11	–
Vietnam^55,d^	2002–2004	Ho Chi Minh	72	11	11	–	3	–	–	–	–	–	–
Thailand^23^	1987–1988	Khon Kaen	113	7	0	6	7	3/17^a^	2	–	–	–	–
Thailand^21^	1998–2001	Bangkok	147	–/22^c^	3	3	10	4/1^a^	7	16	5	–	–
Thailand^46,d^	2001–2002	Bangkok	47	–	–	–	–	–	15	9	4	–	–
Thailand^50^	2001–2002	Khon Kaen	254	11	4	4	10	5/11^a^	4	9	4	–	–
Thailand^48^	2003–2005	Two provinces	1023	–/7^e^	–	–	–	–	1^e^	5^e^	4^e^	23	8^b^
Thailand^28^	2006–2008	Chiang Mai	90	1	6	3	4	10/1^a^	–	–	–	10	2
Thailand weighted average			1674	8/12^c^	3	4	9	5	2	7	4	22	–
Malaysia^40^	1997–1999	Kuala Lumpur	127	6	6	2	10	7/2^a^	3	–	–	–	–
Malaysia^30^	1999–2000	Penang	98	3/3^c^	1	5	7	12	1	–	0	–	15
Malaysia^41^	2000–2002	Kuala Lumpur	346	4	3	4	11	8/1^a^	9	4	6	–	5
Malaysia^46,d^	2001–2002	Kuala Lumpur	112	–	–	–	–	–	12	11	4	–	–
Malaysia^43^	2002–2003	Seremban	108	–	–	–	12	6	–	–	–	–	10^b^
Malaysia weighted average			791	4/4^c^	4	4	10	9	7	6	4	–	7
Singapore^33^	1991–1991	Singapore	96	4	5	4	3	4/2^a^	5	–	0	–	21
Singapore^46,d^	2001–2002	Singapore	73	–	–	–	–	–	10	0	3	–	–
Singapore^55,d^	2002–2004	Singapore	96	6	3	–	0	–	–	–	–	–	–
Singapore weighted average			265	5	4	–	2	–	7	–	1	–	–
Indonesia^46,d^	2001–2002	Jakarta/ Surabaya	37	–	–	–	–	–	3	0	3	–	–
The Philippines^49^	1993–1993	Manila	48	13	19	0	0	8	–	–	–	–	0
The Philippines^46,d^	2001–2002	Manila	136	–	–	–	–	–	7	6	17	–	–
Philippines^55,d^	2002–2004	Manila	55	11	20	–	11	–	–	–	–	–	–
Philippines weighted average			239	12	19	–	6	–	–	–	–	–	–
India^45^	1987–1988	Delhi	46	2	0	2	22	15	–	–	–	–	2
India^25^	1997–1998	New Delhi	60	–	–	–	–	–	–	–	15	–	–
India^26^	1998–2000	New Delhi	42	14	10	12	17	19	24	–	–	–	–
India^52^	2010^f^	Srinagar	100	1	–	7	3	17	–	–	–	–	0
India^22^	2000–2001	Shimla	70	27	–	13	17	16	11	–	–	–	–
India^55,d^	2002–2004	Vellore	104	10	2	–	8	–	–	–	–	–	–
India^47^	2006^f^	Ludhiana	233	9	0	3	3	15	16	18	–	–	5
India^56^	2006^f^	Vellore	80	–	–	–	–	–	–	6	–	–	–
India^53^	2004–2006	Chennai	75	0	0	8	15	0	3	–	–	–	–
India^36^	2005–2008	New Delhi	113	–	–	–	–	–	–	–	2	–	–
India weighted average			923	9	1	6	9	14	14	15	6	–	3
All studies unweighted average			9352	12	7	4	6	4	8	7	3	10	7

GNB: Gram-negative bacilli; – indicates data not available.

Percentages refer to the proportion with a specific pathogen identified. Patients may have more than one pathogen identified.

Country totals are given for hospitalised CAP and are a weighted average of those studies that report data for each organism.

Averages given for all studies (outpatient, hospitalised or severe) are unweighted as variation between countries is likely to be greater than variation due to random error.

^a^ Includes Enterobacteriaceae and non-fermenters but not *Haemophilus influenzae*, *Moraxella catarrhalis* or atypical pathogens. Isolates of *Burkholderia pseudomallei*, when present, are not included in the total but are given separately after a forward slash, and are not included in averages as incidence is geographically variable.

^b^ Cases reported but protocol did not indicate systematic testing. Not included in averages.

^c^ Excluding/including urine antigen testing. If only one value is given, antigen testing was not performed.

^d^ Country-specific data from a multinational study.

^e^ Study used urine antigen testing alone with no culture.

^f^ Period of study not available, therefore publication date given.

**Table 2.** Aetiology of community-acquired pneumonia (CAP) in those treated as outpatients

Country	Year	Site	No. of patients	% *Streptococcus pneumoniae*	% *Haemophilus influenzae*	% *Staphylococcus aureus*	% *Klebsiella pneumoniae*	% Other GNB^a^	% *Mycoplasma pneumoniae*	% *Chlamydophila pneumoniae*	% *Legionella* spp.	% Viruses	% *Mycobacterium tuberculosis*
Japan^20^	1998–2003	Kurashiki	106	12	5	1	0	0	27	11	0	2	–
China^18^	2008–2009	Beijing	197	4/5^b^	1	0	2	2	32	–	0	15	1^c^
Thailand^21^	1998–2001	Bangkok	98	–/13^b^	1	0	0	0	30	37	8	–	–
Thailand^19^	2003–2004	Khon Kaen	44	–/27^b^	32	2	9	9	2	23	7	–	–
All studies unweighted average			445	8/14^b^	10	1	3	3	23	24	4	8	–

GNB: Gram-negative bacilli; – indicates data not available.

Percentages refer to the proportion with a specific pathogen identified. Patients may have more than one pathogen identified.

Country totals are given for hospitalised CAP and are a weighted average of those studies that report data for each organism.

Averages given for all studies (outpatient, hospitalised or severe) are unweighted as variation between countries is likely to be greater than variation due to random error.

^a^ Includes Enterobacteriaceae and non-fermenters but not *Haemophilus influenzae*, *Moraxella catarrhalis* or atypical pathogens. Isolates of *Burkholderia pseudomallei* are not included but are given separately after a forward slash, and are not included in averages as incidence is geographically variable.

^b^ Excluding/including urine antigen testing. If only one value is given, antigen testing was not performed.

^c^ Cases reported but protocol did not indicate systematic testing. Not included in averages.

**Table 3.** Aetiology of community-acquired pneumonia (CAP) in those with severe disease (includes retrospective studies)

Country	Year	Site	No. of patients	% *Streptococcus pneumoniae*	% *Haemophilus influenzae*	% *Staphylococcus aureus*	% *Klebsiella pneumoniae*	% Other GNB^a^	% *Mycoplasma pneumoniae*	% *Chlamydophila pneumoniae*	% *Legionella* spp.	% Viruses	% *Mycobacterium tuberculosis*
Japan^65,b^	1995–2002	Toyama	72	14	3	3	7	11	–	–	3^c^	–	3^c^
Taiwan^58,b^	2001	N Taiwan	169	2	3	4	11	21	–	–	–	–	–
Taiwan^64^	2001–2003	Taipei	62	15	3	10	8	16	2^c^	–	2^c^	2^c^	–
Taiwan^63^	2002–2003	Taichung	22	14	5	5	9	18	0	5	0	–	–
Thailand^61,b^	1999–2001	Khon Kaen	105	13	8	4	11	3/19^a^	–	–	1^c^	–	–
Singapore^62,b^	1989–1993	Singapore	57	7	0	9	9	5/18^a^	7^c^	–	4^c^	–	16^c^
Singapore^59,b^	1991–1993	Singapore	59	5	8	7	15	17/7^a^	2^c^	–	3^c^	–	–
Singapore^60,b^	2003–2005	Singapore	80	13	1	1	5	5	–	–	1^c^	–	4^c^
All studies unweighted average			626	10	4	5	9	12	–	–	–	–	–

GNB: Gram-negative bacilli; – indicates data not available.

Percentages refer to the proportion with a specific pathogen identified. Patients may have more than one pathogen identified.

Country totals are given for hospitalised CAP and are a weighted average of those studies that report data for each organism.

Averages given for all studies (outpatient, hospitalised or severe) are unweighted as variation between countries is likely to be greater than variation due to random error.

^a^ Includes Enterobacteriaceae and non-fermenters but not *Haemophilus influenzae*, *Moraxella catarrhalis* or atypical pathogens. Isolates of *Burkholderia pseudomallei* are not included but are given separately after a forward slash, and are not included in averages as incidence is geographically variable.

^b^ Retrospective study of severe CAP.

^c^ Cases reported but protocol did not indicate systematic testing. Not included in averages.

The aggregated results from all Asian studies of hospitalised patients are compared with a review of European studies of CAP in Table 4. As most of the data are from hospitalised patients, the results discussed below refer to this group unless otherwise stated.

**Table 4.** Aetiology of hospitalised community-acquired pneumonia in Asian and European studies

Organism	Asian studies % (n=38)^a^	European studies^b^ % (n=23)^a^
*Streptococcus pneumoniae*^c^	13.3	25.9
*Haemophilus influenzae*	6.9	4.0
*Mycoplasma pneumoniae*	8.3	7.5
*Chlamydophila pneumoniae*	6.9	7.0
*Legionella* spp.	3.0	4.9
*Staphylococcus aureus*	4.0	1.4
Gram-negative enteric bacteria^c^	9.0	2.7
Viruses	9.8	10.9

^a^ Percentages for each pathogen are the unweighted averages from all of the studies that included diagnostics for that pathogen.

^b^ From Woodhead.^2^

^c^ Gram-negatives are Enterobacteriaceae only, and *S. pneumoniae* includes urine antigen-positives.

#### Streptococcus pneumoniae

*Streptococcus pneumoniae* was the most commonly identified pathogen (13.3% with urine antigen testing included) (Table 1). The proportion of CAP attributed to *S. pneumoniae* showed great variation between countries, with average rates of 24% in Japan, 14% in South Korea and Taiwan, 12% in The Philippines, 8–9% in Thailand, China and India and 4–5% in Malaysia and Singapore. Seven inpatient studies used urine pneumococcal antigen testing, with positive rates of 0–3% using the Wellcogen test and 7–33% using the BinaxNOW test.^21,24,30,35,38,48,57^ The proportion of *S. pneumoniae* isolated from a sterile site was 2.7% in the 14 studies that reported these data versus 10.4% in those who had positive cultures from any site in these studies.

#### Haemophilus influenzae

The overall rate for Asia was 6.9%, with variation between Asian countries (Table 1). The highest rates were in The Philippines (19%), followed by Japan (10%) and China (9%). The lowest rates were found in India and South Korea (both 1%). In The Philippines, *H. influenza*e was found more commonly than *S. pneumoniae*. The proportion with *H. influenzae* isolated from a sterile site was 0.1% in the 12 studies that reported these data versus 4.5% in those who had positive cultures from any site in these studies.

#### Mycoplasma pneumoniae and Chlamydophila pneumoniae

Twenty-nine studies included diagnostics for these organisms; all used serology and four also used PCR. *Mycoplasma pneumoniae* and *C. pneumoniae* were common CAP pathogens (identified in 8.3% and 6.9% of all hospitalised patients, respectively) (Table 1), with higher rates in outpatient cohorts (22.9% for *M. pneumoniae* and 23.6% for *C. pneumoniae*) (Table 2).

#### Legionella spp

The proportion of patients diagnosed with *Legionella* spp*.* was low (3.0%). One multicentre study described a particularly high rate of infection at a study site in Manila (17%),^46^ but there are no other data from The Philippines to corroborate this. In one study, the proportion with *L. pneumophila* detected was 2.7% by urine antigen, rising to 6.2% with serology.^46^ Another study found no *L. pneumophila* urine antigen-positive cases among 373 patients, but detected *Legionella longbeachae* infection in 3.8% using serology and PCR.^48^

#### Gram-negative bacilli (GNB)

GNB were identified in 13.0% of hospitalised patients when averaging all Asian studies (Table 1; see footnote for definition). The lowest proportions were seen in East Asia, with a higher number reported in Southeast Asia and India. The most common GNB isolated was *Klebsiella pneumoniae* (6.3%), largely from sputum, but the proportion with *K. pneumoniae* bacteraemia averaged 2.0% in the 17 studies where this was reported, with an overall isolation rate of 6.2% in these studies.

A comparison of studies performed in different settings revealed higher rates of GNB with increasing severity (Table 5). Among patients with severe CAP, GNB were the most common pathogens identified (21.5%).

**Table 5.** Aetiology of community-acquired pneumonia by study setting

Organism	Outpatient % (n=4)^a^	Inpatient % (n=38)^a^	Severe % (n=8)^a^
*Streptococcus pneumoniae*	14.3	13.3	10.3
*Haemophilus influenzae*	9.5	6.9	3.9
*Mycoplasma pneumoniae*	22.9	8.3	–
*Chlamydophila pneumoniae*	23.6	6.9	–
*Legionella* spp.	3.7	3.0	–
*Staphylococcus aureus*	0.8	4.0	5.1
Gram-negative bacteria^b^	2.9	13.0	21.5
Viruses	8.3	9.8	–

– Indicates data not available.

^a^ Percentages for each pathogen are the unweighted averages from all of the studies that included diagnostics for that pathogen in outpatient, inpatient and intensive care unit settings.

^b^ All Gram-negative bacteria included.

In two studies carried out in northeast Thailand, *Burkholderia pseudomallei* was the most common pathogen identified in CAP and was cultured in 15% of 367 cases. Studies of patients hospitalised with CAP in Malaysia and Singapore identified *B. pseudomallei* in 1–2%, although the proportion was much higher among patients admitted to the intensive care unit in Singapore (9.2%) (Table 3). A prospective Cambodian study of inpatient CAP found *B. pseudomallei* in 1.6% of patients (not included in the quantitative review as there were no data for other pathogens).^66^

#### Staphylococcus aureus

*Staphylococcus aureus* was isolated in 4.0% of patients, increasing to 5.1% in patients with severe CAP (Table 5). The proportion with *S. aureus* isolated from a sterile site was 1.9% in the 14 studies that reported these data versus 4.5% in those who had positive cultures from any site in these studies.

#### Mycobacterium tuberculosis

Ten studies included TB diagnostics in their protocol; all used microscopy and four also performed culture. Rates of *M. tuberculosis* in patients presenting with CAP were >10% in three of these studies (Table 1). Six other studies reported some cases with TB but did not test systematically.

####  Moraxella catarrhalis

*Moraxella catarrhalis* was isolated in <1% of hospitalised patients on average (data not shown).

## Discussion

This review represents a synthesis of published Asian CAP aetiology studies and reveals several important patterns. In the West, GNB and *S. aureus* are uncommon causes of CAP and are usually found in the context of hospital-acquired pneumonia (Table 4).^2,67,68^ In Asia these organisms were identified in a higher proportion of patients. Conversely, although *S. pneumoniae* was commonly identified, it was relatively less important than in most European studies. Also, a substantial proportion of patients presenting with CAP in Asian countries were found to have TB, which is often considered to cause only more chronic pulmonary disease. Finally, *B. pseudomallei* was a major cause of CAP in northeast Thailand and was also reported in other Southeast Asian countries.

### Limitations

There were several challenges in conducting this review, resulting in some limitations to the data. First, there were limitations in determining aetiology using currently available methods. With the exception of *B. pseudomallei*, interpretation of the clinical relevance of GNB such as *K. pneumoniae* is problematic as most are isolated from sputum culture and could therefore represent colonisation rather than infection. While this is also true for Gram-positive pathogens such as *S. pneumoniae* and *S. aureus*, pre-sampling antibiotic use favours colonisation by GNB. Obtaining reliable results from sputum culture is difficult, even in well-resourced hospitals. A lack of rigorous microbiological standards, for example specimen collection following antibiotic use, delays in specimen transport, or culture of specimens with inadequate microscopic screening for white blood cells, might be expected to reduce isolation of more fastidious bacteria such as *S. pneumoniae* and *H. influenzae* and to increase isolation of Enterobacteriaceae and *S. aureus*.^9^ The most common GNB isolated from sputum in most studies is *K. pneumoniae*, and while this is a well-recognised CAP pathogen, the high rate of isolation of *K. pneumoniae* could also relate to the issues outlined above. In some cases, isolation of GNB almost certainly represented colonisation rather than infection. One such example is an Indian study of 46 patients that reported Gram-negative pneumonia in 37% based on sputum culture, most of whom responded to penicillin despite the organisms being intrinsically resistant to this antibiotic.^45^ Without routine collection of invasive respiratory specimens it is difficult to have full confidence in the clinical relevance of culture results. For example, we are aware of only one large study in the past 20 years, performed in Kenya, which used routine transthoracic needle aspiration for bacteriological diagnosis of CAP.^69^ In this study of hospitalised patients, only 0.7% of HIV-negative patients had a GNB isolated compared with an average of 7.9% in three other comparable African studies that used sputum culture.^70–72^ It is notable that where data were available, comparison of samples positive from sterile sites with samples positive by any means was similar for *S. aureus* (1.9% vs. 4.5%), *S. pneumoniae* (2.7% vs. 10.4%) and *K. pneumoniae* (2.0% vs. 6.2%), while *H. influenzae* was less commonly isolated from sterile sites (0.1% vs. 4.5%). However, whether this reflects that this bacterium was more commonly a contaminant of sputum or differences in pathogenesis or virulence is difficult to determine.

A second limitation is the absence of data from large parts of Asia (Figure 2). The data included in this review largely come from patients admitted to large urban hospitals in relatively wealthy countries. Consequently, there are few data from areas were the highest burden of disease is expected. A limitation of the methodology is that only studies published in English were included and therefore relevant studies in other languages will have been missed. There were seven studies identified from the database and reference searches that might have been included if published in English, but these were all from Japan, China or South Korea, countries that already have 19 studies included here.

#### Gram-negative bacilli

Despite these issues, the higher isolation rate of GNB in CAP cannot be discounted. Many studies that reported high rates of GNB in sputum have no apparent methodological deficiencies compared with Japanese or western studies describing much lower rates. Furthermore, the proportion of patients with bacteraemic *K. pneumoniae* pneumonia, where there can be little doubt of its clinical relevance, was 1.7% in the studies that reported it compared with only 0.1% in two large western studies.^73,74^

The underlying cause of the higher rate of GNB in Asian studies is unclear; importantly, those studies that quantified prior antibiotic use provided no evidence to suggest that this was responsible for the higher rates of sputum isolation of *K. pneumoniae*. Although there are few data comparing GNB carriage internationally, two studies report high rates (36–57%) of GNB nasopharyngeal carriage in developing countries and lower rates of 4–9% in developed countries.^75,76^ If confirmed, geographical differences in the nasopharyngeal microbiome might account for higher rates of Gram-negative CAP in Asia, although this could also cause more Gram-negative contamination of sputum samples. Other explanations for the high rates of Gram-negative CAP described could relate to underlying diseases, with the unequivocally high rates of Gram-negative pneumonia seen in malnourished children as an extreme example.^77^ A syndrome of invasive *K. pneumoniae* infection, which can include pneumonia and liver abscess, is well described in Taiwan and several other Asian countries, although the cause for this geographic distribution is unknown.^78–82^

*Burkholderia pseudomallei* is well recognised as a major cause of community-acquired septicaemia and pneumonia in northeast Thailand. Although less common, it is also important in other Southeast Asian countries because of the high mortality rate and implications for antibiotic selection. No infection was identified in studies outside Southeast Asia, although it has been described in case series from other Asian countries such as India and Taiwan, and it can be overlooked if unexpected.^83^

#### Gram-positive cocci

The proportion of CAP with *S. aureus* followed a similar, although less marked, geographical pattern to that of GNB and the same uncertainties apply. With the exception of Japan, the proportion of CAP patients diagnosed with *S. pneumoniae* was lower than in western studies. Issues of microbiological technique may have some impact but this should not affect urine antigen positivity, which is substantially different between one large Thai study (7%) compared with similarly well-designed Japanese (33%) and Spanish (31%) studies.^15,35,48^ While higher rates of infection with other pathogens would lower the proportion attributable to *S. pneumoniae*, this seems insufficient to explain the observed differences.

#### Mycobacterium tuberculosis

Many studies found that TB was common among patients presenting with CAP, which is also the case among high-prevalence populations in Africa, the Middle East and the USA.^69,70,84–86^ However, because of the inconsistent approach to TB diagnosis the data are incomplete, making comparisons between countries difficult. Despite these uncertainties, these data show that *M. tuberculosis* is an important cause of lower respiratory infection in many parts of Asia. Decisions about the choice of routine investigations performed for CAP and the choice of empirical treatment should reflect this. In particular, care should be taken with the empirical use of antibiotics with antituberculous activity, such as quinolones, which may lead to a partial response, mask diagnosis and ultimately promote the development of drug-resistant TB.

#### Atypical bacteria

The number of *M. pneumoniae*, *C. pneumoniae* and *Legionella* infections varied greatly. This may have related to geographical variation or to changes in incidence during different study periods, as studies typically lasted only 1–2 years (shorter than the classic epidemic cycle of *M. pneumoniae*). Furthermore, there was wide variation in diagnostic techniques, with different methods and diagnostic criteria in almost every study. Use of a simultaneous control group was rarely performed. The gold standard for serological diagnosis of a rise in antibody titre between acute and convalescent serum specimens was widely used in East Asia, although diagnoses were also often made on single high IgM titres. Repeat specimens were not used at all in most of the Indian studies, presumably as it was not practical to obtain them.

### Conclusions

Appropriate selection of diagnostic tests and empirical treatment for CAP is crucial and depends both on knowledge of the common pathogens identified in aetiology studies and on the results of therapeutic trials. The available aetiology data from Asia are limited but suggest that use of empirical guidelines based on western data may be inappropriate due to the higher proportion of CAP associated with GNB and TB. Insufficiently broad antibiotic coverage when treating Gram-negative pneumonia may result in avoidable morbidity and mortality, whereas excessive use of broad-spectrum antibiotics increases healthcare costs for those least able to afford it and is already contributing to an unprecedented problem of antibiotic resistance in Asia.^87^

Future aetiology studies should be large, have rigorous microbiological methodology (including routine testing for *M. tuberculosis*) and include less developed or rural areas with little existing data. Interpretation of results is difficult without the routine collection of invasive specimens, such as transthoracic needle aspiration, and the inclusion of a control group. The multicentre Pneumonia Etiology Research for Child Health case–control study includes all of these elements and will make a major contribution to understanding childhood pneumonia.^88^ A similarly rigorous study is needed in adults. An additional approach to inform treatment is via randomised comparisons of different empirical treatment strategies. Until now there have been relatively few trials comparing therapeutic regimens for adult CAP in Asia. By avoiding the need for non-routine diagnostics, perhaps other than a sputum sample for TB, it may be possible for a trial to extend to areas where aetiology studies are less practical. Such trials could provide reliable data to guide the empirical treatment of CAP across Asia, home to one-half of the world's population.
